# A Systematic Review of Genes Affecting Endocochlear Potential

**DOI:** 10.1007/s10162-026-01046-y

**Published:** 2026-03-19

**Authors:** Darcey A. Kirwin, Morag A. Lewis, Karen P. Steel

**Affiliations:** 1 Wolfson Sensory, Pain and Regeneration Centre, King’s College London, London SE1 1UL, UK

**Keywords:** Stria vascularis, Endocochlear potential, Genetics, Mouse, Deafness

## Abstract

**Purpose:**

Hearing loss is highly heterogeneous. Any one of hundreds of genes and dozens of cell types can be involved in the pathological processes in the auditory system. One class of hearing loss results from a reduction of the endocochlear potential (EP), a voltage maintained in the endolymph that bathes the upper surface of the sensory hair cells in the cochlea. Understanding the landscape of genes involved in reduced EP will be useful in developing targeted therapies for this type of hearing loss. Here we review these genes.

**Methods:**

Research articles that report genes impacting EP in mutant mice were collated using several different approaches. Cell type-specific expression and patterns in their biological function were investigated.

**Results:**

We report 55 genes associated with reduced EP as well as 43 genes shown to underlie deafness but with no change in EP. We show that of these 55 reduced EP genes, 27 are linked to deafness in humans and therefore these patient populations are candidates for having a reduced EP. We demonstrate that the expression of reduced EP genes is not clustered to a particular cell type within the stria vascularis or organ of Corti.

**Conclusion:**

This analysis highlights the broad range of expression patterns and functions of genes involved in generating and maintaining the mammalian EP. Furthermore, the lists presented here can inform the direction of translational research for different forms of human hearing loss.

## Introduction

Hearing impairment is a prevalent disorder, and onset can range from before birth to late adulthood. Childhood deafness affects one in 1000 children and increases until over one in every two adults above 70 experience a form of deafness [1]. Hearing impairment is a heterogenous disorder where environmental factors including noise, chemicals and heavy metal exposure can contribute but genetics is a major cause. To date, 156 genes have been identified underlying non-syndromic deafness in humans [1]. An unknown proportion of these genes may be associated with a reduced endocochlear potential (EP) as a primary cause of the onset of deafness.

EP is a high resting potential of around + 100 mV maintained in the endolymph of scala media of the cochlea. It is an essential component for normal hearing because the EP provides an electrochemical driving force for cations to enter the hair cell when mechanoelectrical transduction channels are opened in stereocilia bundles by sound [2]. Once the EP is reduced or abolished, the function of cochlear hair cells is impaired, leading to hearing impairment.

The EP is produced and maintained by the stria vascularis (SV), a highly specialised and vascularised tissue located in the cochlear lateral wall where it performs a critical function in maintaining cochlear homeostasis. The SV is mainly composed of marginal, intermediate and basal cells along with other cell types associated with its capillaries such as endothelial cells and pericytes [3, 4]. A number of critical ion transport proteins in the SV actively pump potassium into the endolymph-filled scala media (reviewed elsewhere [3, 5]). Together, these highly metabolic processes maintain the endolymph at a high potassium (~150 mM), low sodium (~1.3 mM) state. This contrasts with the low potassium (6.0 mM), high sodium (140 mM) composition of perilymph [2].

Much of the potassium pumped into scala media comes from recycled potassium [4, 6–8]. Potassium passing through the transduction channels of hair cells is removed via their basolateral membranes into the supporting cell network where it passes through gap junctions to the fibrocytes of the spiral ligament and to the basal and intermediate cells of the stria vascularis. There it is actively pumped out by channels on intermediate cells via the marginal cells into scala media at a rate that allows the development of the endocochlear potential. The boundaries of scala media need to provide an electrical resistance for EP to develop. A high potassium concentration alone is insufficient to generate EP, as illustrated for example by the dissociation between high scala media potassium concentrations and low potential recordings in the *Slc26a4* mouse mutant [9].

Understanding which genes are involved in generating endocochlear potential will be critical for developing targeted therapies for this class of hearing loss. This review aims to identify all known genes involved in EP production and analyse patterns of expression and biological functions in the mammalian inner ear.

## Methods

### PRISMA Search Strategy

**Fig. 1:**
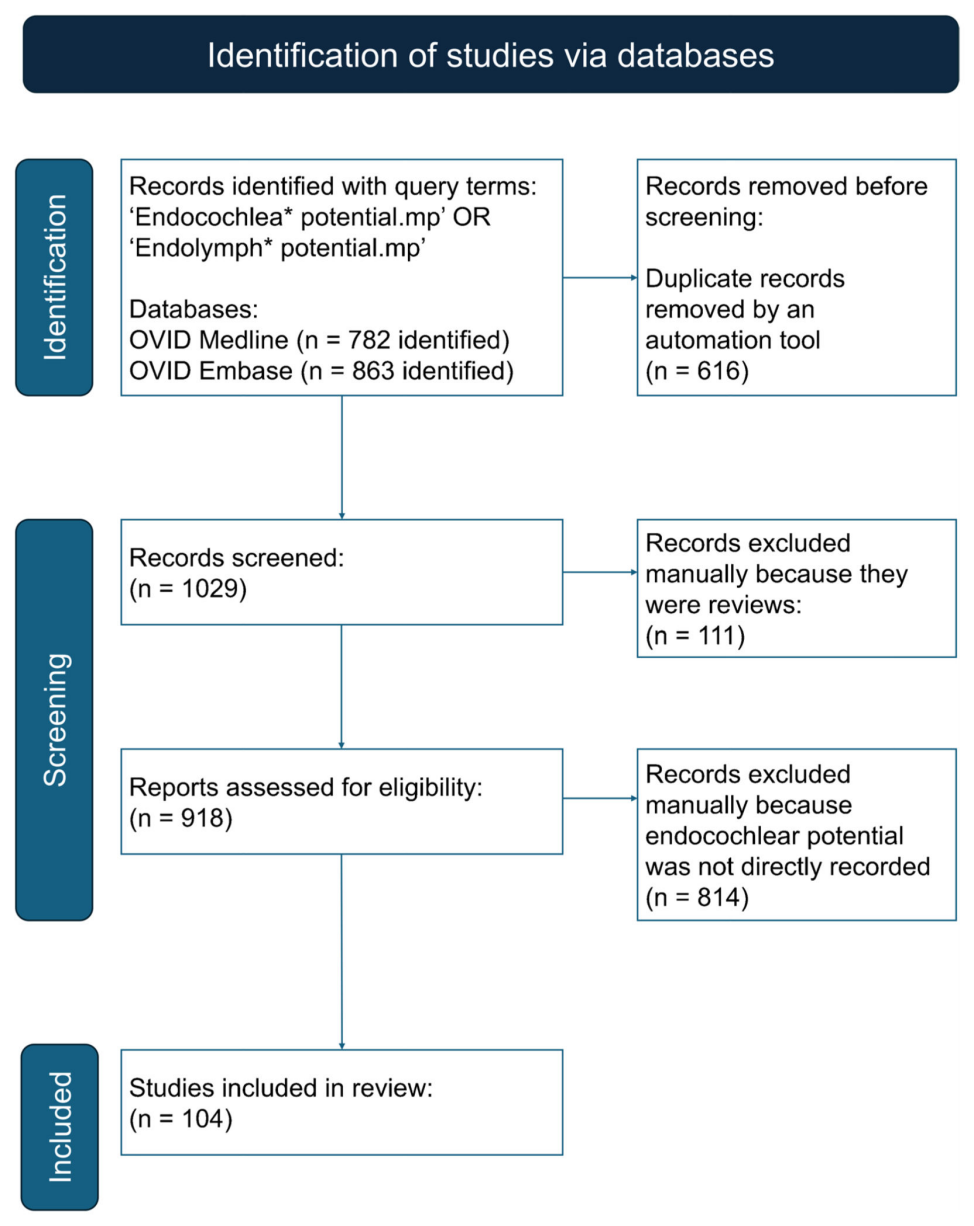
PRISMA flowchart detailing the inclusion and exclusion criteria of articles used in this review for retrieving papers using the PRISMA search strategy

The PRISMA flowchart was followed for this review [10]. Database searches were retrieved for OVID Medline on 14th February 2024 and for OVID Embase on 21 st February 2024. The query terms used for both databases were ‘Endocochlea* potential.mp’ OR ‘Endolymph* potential. mp’. Initially, following paper retrieval from both databases a v lookup table function was used in Microsoft Excel to remove duplicate documents between the two database search results. This was the only automation tool used throughout this section of the systematic review. A second reviewer also performed this screen independently to make sure reports removed due to being duplicates were accurate. Following this, the remaining papers were screened to remove review articles manually. Subsequently, a detailed analysis was performed on the remaining articles to extract information relevant to this systematic review (Fig. 1). A Microsoft Excel spreadsheet was populated with relevant information including EP and the anaesthetic agent used for EP recordings. All results were included in the EP gene list unless stated. Reduced EP was defined by mutant EP being significantly lower than control EP in the original research paper. It was not possible to choose one value as a cut-off point for reduced EP because wildtype EP was so variable.

### Annotating Published Deafness Gene List

As some genes affecting EP appeared to be missing from the list of the PRISMA analysis, we hypothesised that papers reporting EP may have been missed due to EP not being mentioned in the title, abstract or keywords. Therefore, a curated gene list of known deafness genes was manually annotated [11]. This list was downloaded on 23rd July 2025. Each gene underwent a search of ‘gene name’ and endocochlear potential using the Google search engine, as well as a second PubMed search using the advanced search feature and the following query term of *Gene name* AND ‘Endocochlea* potential.mp’ OR ‘Endolymph* potential.mp’.

### Searching the Mouse Genome Informatics (MGI) Database

A list of genes annotated with the term “abnormal endocochlear potential” was obtained from the Mammalian Phenotype Ontology Annotations section of MGI (https://www.informatics.jax.org/mp/annotations/MP:0002630, accessed 2nd October 2025).

### Use of AI

As a final method to ensure all primary research papers investigating genes linked to EP were captured, the language learning model (LLM) ChatGPT (OpenAI (2023) August 2025 version) was prompted to search through published literature and provide a list of genes where the EP had been investigated. One prompt was used to retrieve reduced EP genes: ‘Conduct a literature search for any literature which report reduced endocochlear potential or endolymphatic potential in mice. Please provide a reference and Ensembl ID for each source’. To retrieve non-reduced EP genes the following prompt was used: ‘Conduct a literature search for any literature which recorded endocochlear potential or endolymphatic potential in mice. Please provide a reference and Ensembl ID for each source’. To ascertain the effectiveness of these searches we report two figures below. Firstly, we report the hallucination rate, defined as the number of falsely reported genes as a percentage of the total number of genes given by ChatGPT in response to prompts. Secondly, we report the success rate, defined as the number of correctly reported genes as a percentage of the total number of EP genes reported in this review.

### Analysis of Gene Lists in gEAR

Gene names were prepared for entry into the gEAR expression database [12]. Synonyms and Ensembl IDs were listed in a separate excel table to ensure they could be included into downstream analysis accurately. The gEAR databases were used to view the expression of these genes in the mouse inner ear using the multi-gene display feature [12]. Two individual single-cell RNAseq datasets were used to investigate the expression of genes of interest in different cells of the cochlea at different ages, and to look for any sex differences in the expression of these genes [13, 14].

### Analysis of Gene Lists in Gene Ontology (GO) Analysis

GO analysis [15] was used to indicate gene function of the gene lists on a large scale by combining evolutionary data, functional ontology, and statistical analysis tools to view the biological process and molecular function of these genes in mammals. The full list of 55 genes associated with reduced EP, and separately the full list of 43 genes associated with non-reduced EP, were input as a list of their Ensembl IDs and the *Mus Musculus* reference list was selected. A Fisher’s exact test was selected, and the false discovery rate was used to correct for multiple comparisons. Fold enrichment was used to determine how much more frequently a particular gene ontology term or pathway is represented in the EP gene list compared to the reference list, and fold enrichment was used as the primary filter for tables presented below. An overrepresentation test was also used to compare the observed number of genes in a specific functional category to the expected number based on background distribution. This allows an assessment of whether the observed enrichment is significantly different from what would be expected by chance. A small p-value resulting from the Fisher’s exact test indicates that the observed enrichment is likely not due to random chance. GO analysis was performed on the complete gene list on 4th November 2025 using the annotated PANTHER (Protein ANalysis THrough Evolutionary Relationships) 19.0 version released on 16th March 2025.

### Links to Human Deafness

Genes on the derived lists were annotated for any reported links to human deafness by searching each gene in the Online Mendelian Inheritance in Man database (OMIM. org; data retrieved 5th November 2025).

### Control Mouse EP Value vs Anaesthetic

Papers retrieved from the above analysis were examined to annotate the wildtype EP value and anaesthetic agent used. Occasionally, the mean wildtype EP value was not given as text in the figures; for example, the reduction may be expressed as a percentage change, or mV change rather than specific numbers. In these cases, the EP value was estimated based on the graphical presentation of results. If a range of values was reported in the text, the median was taken. If more than one age group was reported, an early adult age group was selected as representative to limit the impact of age as a confounding variable. In a few cases, only heterozygote animals were used as controls and these are included. Details of the reports used are presented in Supplementary Table S1.

## Results

### Each Search Method Varied in Effectiveness for Retrieving EP Papers

Using the PRISMA search strategy, a total of 104 publications were retrieved, which generated a list of 70 different genes with EP measurements (Tables 1, 2). Initially, the list of genes was split into those which did and did not produce a reduced EP at the age of recording. There was a total of 58 genes where disruption to gene expression led to a significant reduction in EP compared to controls. There were fewer examples where EP was recorded but no significant reduction was found compared to controls. A total of 12 genes fit into this category. For some genes, several papers reported EP.

Annotating the manually created list of known deafness genes [11] yielded 10 additional genes with reports of reduced EP (*Atp2b2, Caml, Cdk5, Cldn9, Ednrb, Foxi1, Hmx3, Lmx1a, Tyr* and *Tyrp1*) and 28 additional genes reporting normal EP. We believe this difference in retrieval is due to some results not being mentioned in the title or abstract text or being buried within supplementary material and therefore missed from the PRISMA search. This highlights a limitation of using multipurpose (.mp) database searches such as within Medline or Embase for retrieving literature.

Searching MGI retrieved a total of 29 genes with abnormal EP, of which 24 were correct and already included in our earlier lists plus 5 that were excluded as false positives. The false positives were excluded because: only anoxia EP was abnormal (*Ildr1, Slc12a6*); Reissner’s membrane was collapsed making EP measurement unreliable (*Kcnj10*); EP was higher than normal (*Espn*); two genes both of which needed to be mutated (*Thra, Thrb*).

Finally, ChatGPT was used to retrieve additional reports of genes which were not found by the PRISMA search or other approaches. When prompted to retrieve a list of genes reported to lead to reduced EP, it retrieved a total of 43 genes. From reading these reports, 1 new gene was found (*Ednrb*), 16 were already in the reduced EP list from the PRISMA search and the rest of the genes were incorrect, with either the information provided on the value of EP recorded being wrong or the reference being made up. For the query about non-reduced EP genes, a total of 28 genes were retrieved. Upon manual investigation, 3 new genes were found (*Cib2, Map3k1* and *Tecta*), 6 were already in the non-reduced EP list from the PRISMA search and the remaining 19 genes were hallucinations. This gives an overall hallucination rate of 63% (45 genes out of 71). In addition, considering the genes that were not retrieved by the searches (false negatives), there was a success rate of 27% (26 out of 98). We also note that searching using the same prompt can produce a different response from ChatGPT. This was particularly evident if the paid version is used or the Deep Research feature enabled. This highlights a problem with the reproducibility of retrieving literature using LLMs compared to database searching with strict query terms such as in OVID.

In the lists of genes with reports of EP level in Tables 1, 2, in most cases only one timepoint was selected for EP recordings, so it is possible that some of these mutants would go on to develop a reduction in EP with increased age. As a further refinement to the list, genes which only produced a reduction in EP when present in a multiple knockout were excluded. This included three genes affected by the mutation in the headbobber mutant, *Chst15, Cpmx2* and *Gpr26* [16]; *Slc16a10* and *Slc16a2* [17]; *Thra* and *Thrb* [18] and finally *Phex* and *Sms* [19]. We also excluded genes where a reduction in EP was reported alongside early collapse of Reissner’s membrane. This is because in these cases it is possible that an EP value of close to 0 mV for mutants was due to the electrode being inserted into perilymph rather than into an open scala media. Genes excluded on this basis include *Foxi1, Kcne1, Kcnj10, Kcnq1* and *Lmx1a* [9, 20–23].

Overall, this protocol generated a list of 55 genes in the reduced EP category and 43 genes in the non-reduced EP category (Tables 1, 2).

### Reduced EP Genes are Expressed in Various Cell Types and Expression Does not Vary Much Between Sexes or with Increased Age

A key aim of this review is to understand the expression pattern of reduced EP genes within the cochlea to search for novel trends. To investigate this for the genes in Table 1, we used the multi-gene display feature of gEAR to create heat-maps from existing RNA sequencing datasets of cochlear tissue. Two RNA sequencing datasets were chosen.

**Fig. 2:**
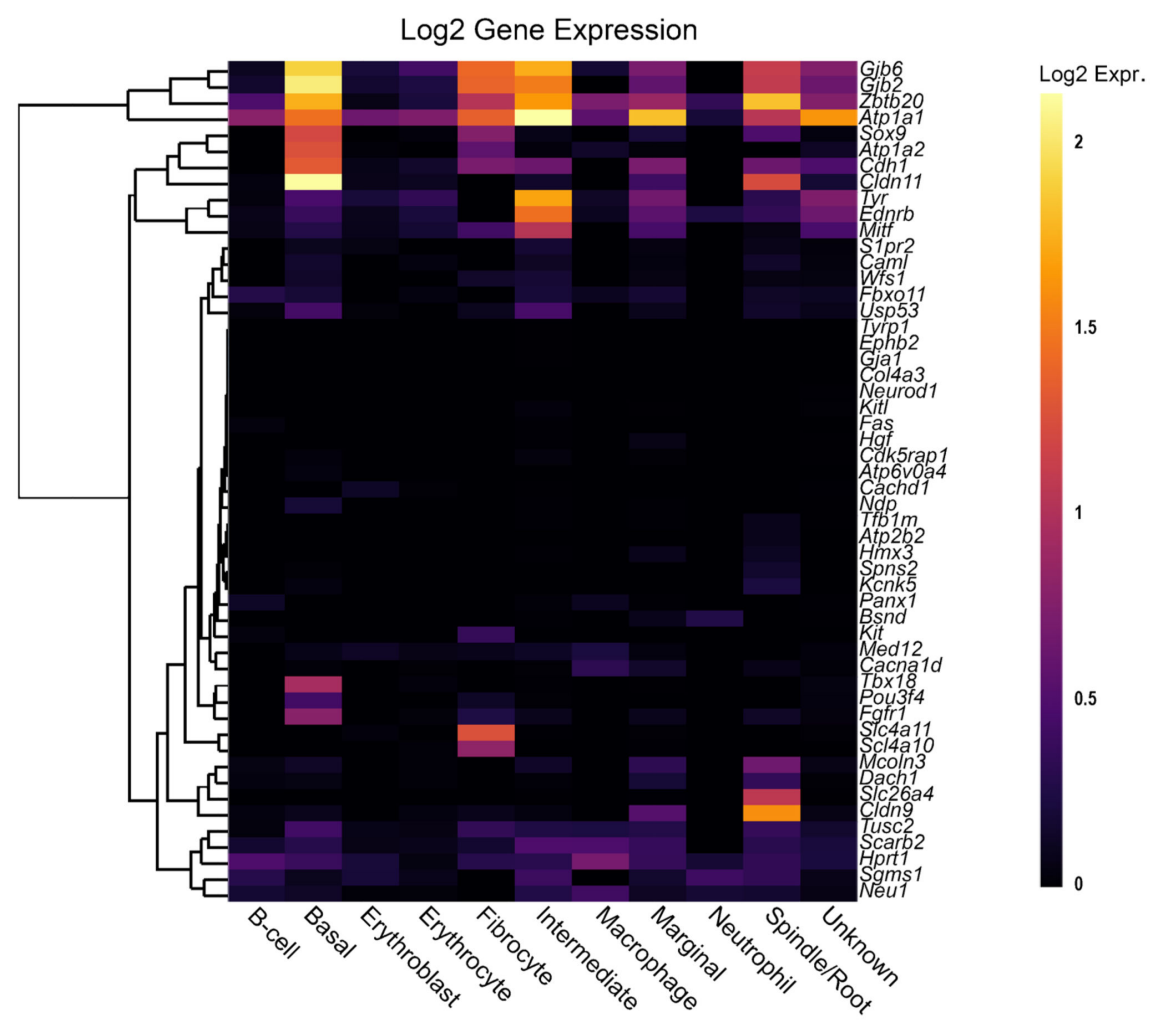
The expression of reduced EP genes in the adult mouse SV. The 55 reduced EP genes were the input for the multi-gene array. The house-keeping gene *Hprt1* was added to the gene list for comparison. Settings were adjusted so that the primary grouping was cell type. Genes were clustered based on a Manhattan (city block) approach. Four genes were not included because they were not found in this dataset (*Cga, Grid1, Hgf* and *Pou1f1*). Source: UMgEAR Portal, [13]

**Fig. 3:**
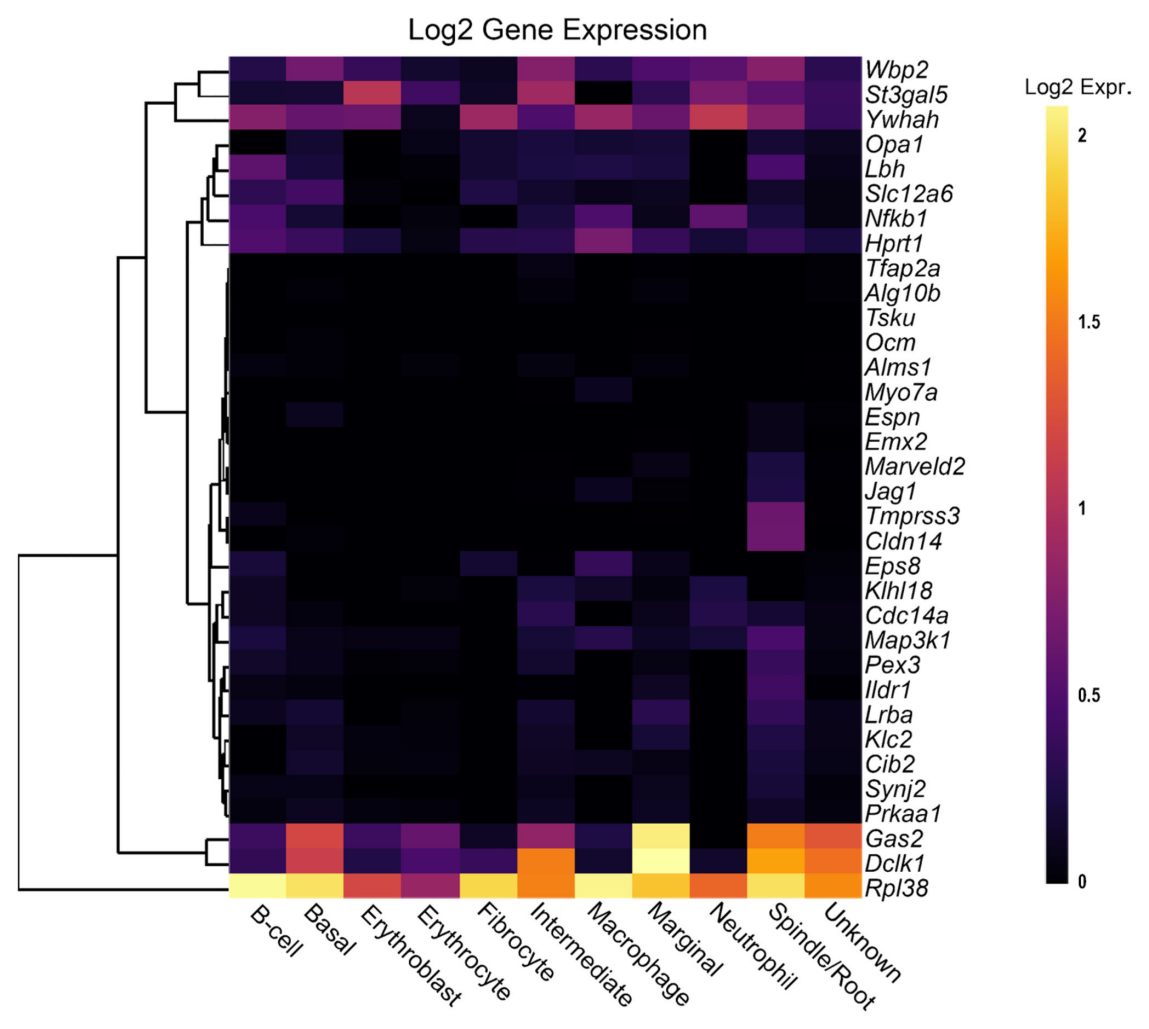
The expression of non-reduced EP genes in the adult mouse SV. The 43 non-reduced EP genes were the input for the multi-gene array. The house-keeping gene *Hprt1* was added to the gene list for comparison. Settings were adjusted so that the primary grouping was cell type. Genes were clustered based on a Manhattan (city block) approach. Source: UMgEAR Portal, [13]

Firstly, expression from single cell RNA-Seq in the adult (P30) CBA/J mouse SV tissue was analysed [13]. The heat-map of reduced EP genes in the adult SV (Fig. 2) reveals that many of these genes are expressed in one or more cell type and the expression of reduced EP genes is quite well distributed between different cell types. Cell types with the most highly expressed reduced EP genes include basal cells, intermediate cells, marginal cells, fibrocytes and spindle cells. Immune and blood cells showed low levels of expression of reduced EP genes. Interestingly, a large proportion of reduced EP genes are not expressed highly or at all in the adult SV. Some of these genes, for example both *Kit* and *Kitl*, are known to be important during developmental stages [24, 25] for the survival and migration of melanocytes [26], the precursors to the intermediate cells of the SV. Other genes in this group with low adult expression may also be highly expressed during developmental stages but not during adulthood and hence not well represented by choosing this dataset. Four out of the 55 input genes could not be added to the heatmap because they were not identified in the original dataset used to analyse this gene list. This dataset was also used to analyse the list of non-reduced EP genes (Fig. 3). Interestingly, we observe high expression of several of these genes in SV cell types including *Gas2, Dclk1* and *Rpl38*. This highlights that for some genes expressed in the SV, disruption to expression does not always lead to reduced EP.

**Fig. 4:**
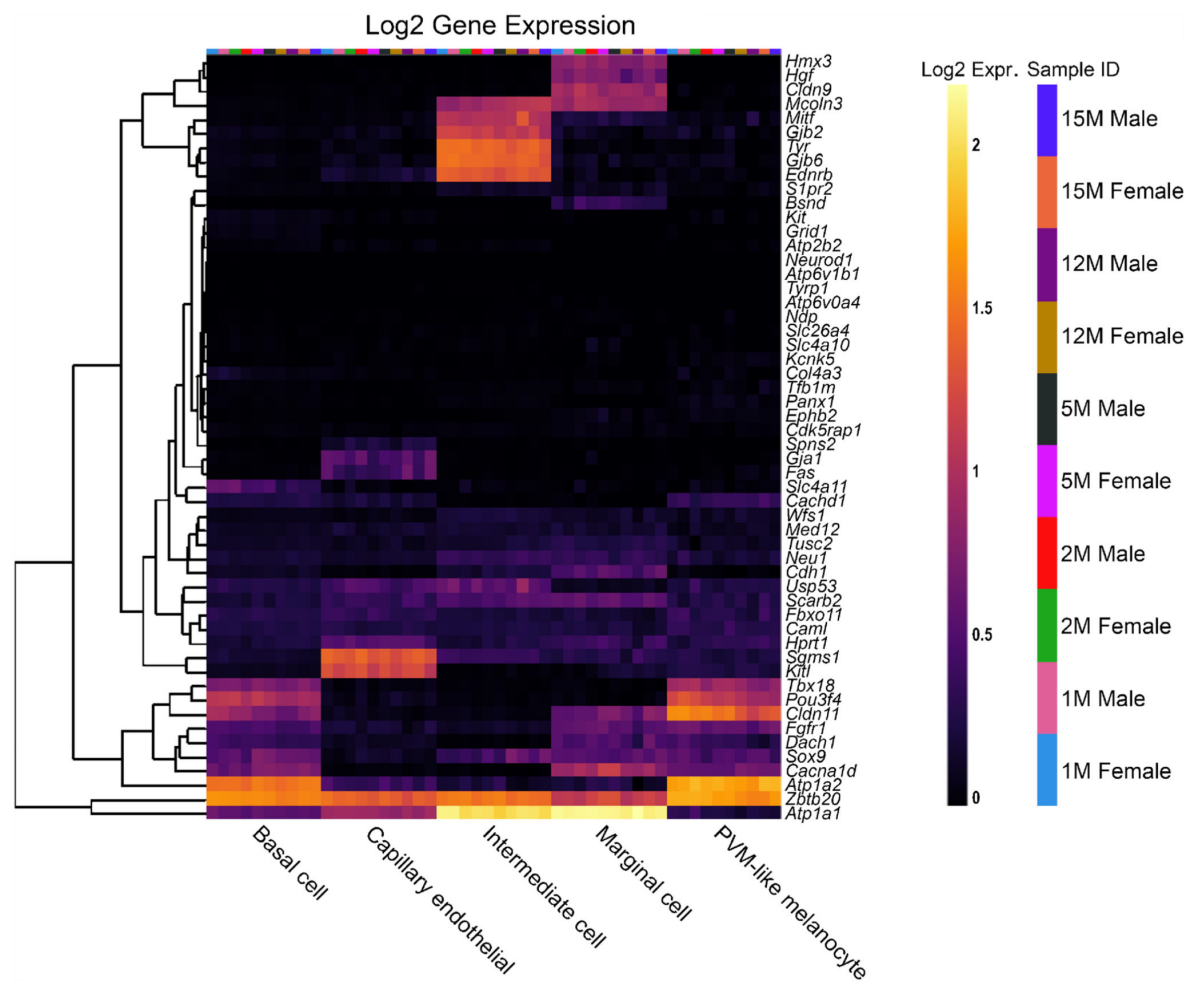
The expression of reduced EP genes in SV cell types between 1 and 15 months of age. The 55 reduced EP genes were the input for the multi-gene array. The housekeeping gene *Hprt1* was added to the gene list for comparison. Settings were adjusted so that the primary grouping was cell type. Genes were clustered based on a Manhattan (city block) approach. Within each gene and cell-type category, samples are presented in increasing age from left to right, with females and males within the same age group adjacent to each other. PVM = perivascular macrophage. Source: UMgEAR Portal, [14]

To compare the effects of sex as well as increasing age within the SV specifically, a second single-cell RNA-Seq dataset was used to analyse the reduced EP gene list, from C57BL/6 J mice [14] (Fig. 4). For most reduced EP genes, the expression throughout adult life is consistent, although there are some examples of expression changing with age. For example, *Slc4a11* expression decreases with age in basal cells, whereas *Spns2* and *Sox9* expression increases with age in endothelial and intermediate cells respectively. There are no clear, consistent trends regarding sex differences in the reduced

**Fig. 5:**
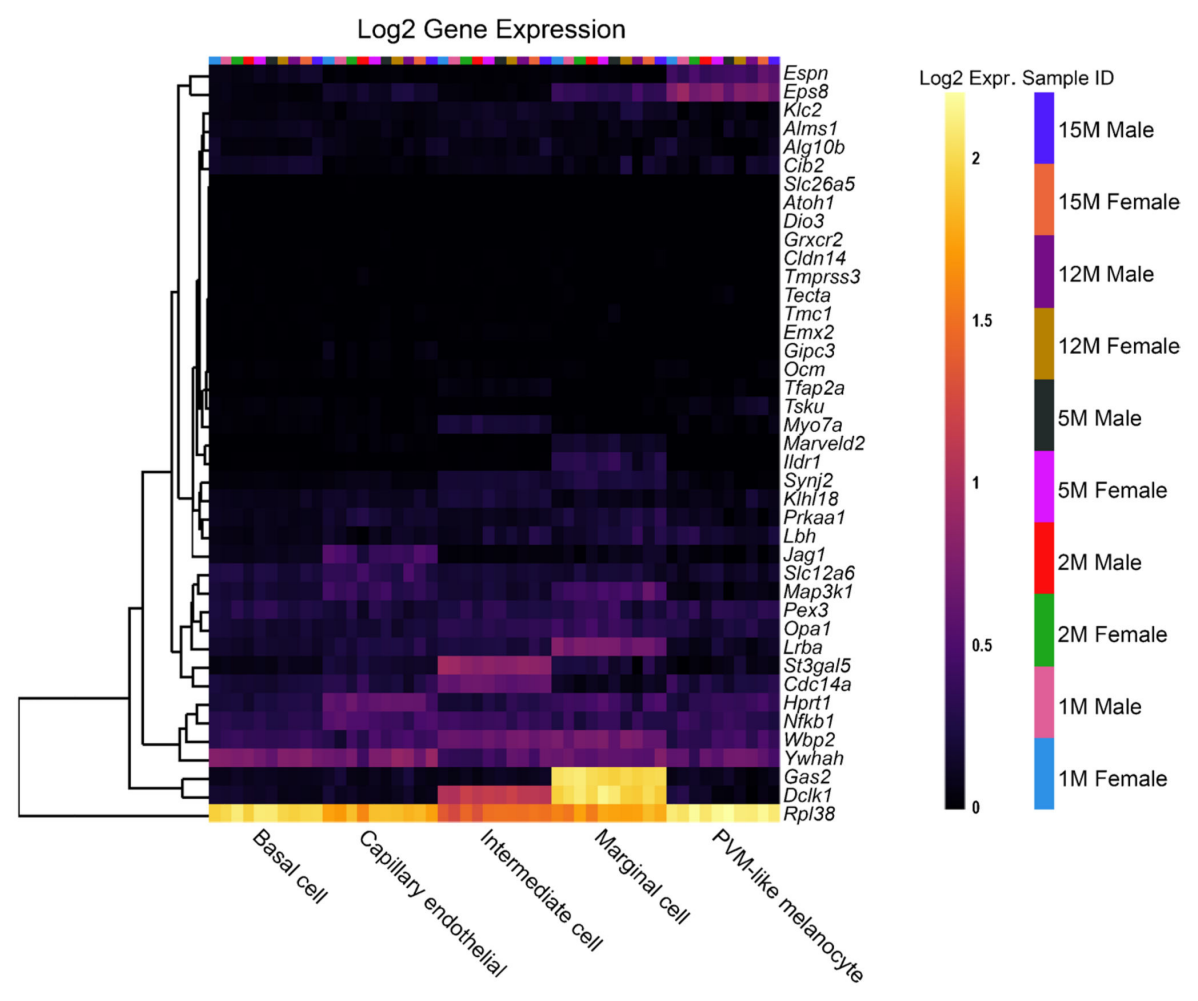
The expression of non-reduced EP genes in SV cell types between 1 and 15 months of age. The 43 non-reduced EP genes were the input for the multi-gene array. The house-keeping gene *Hprt1* was added to the gene list for comparison. Settings were adjusted so that the primary grouping was cell type. Genes were clustered based on a Manhattan (city block) approach. Within each gene and cell-type category, samples are presented in increasing age from left to right, with females and males within the same age group adjacent to each other. PVM = perivascular macrophage. Source: UMgEAR Portal, [14]

EP gene list analysed using this dataset. When analysing the non-reduced EP gene list in this scRNAseq dataset, a similar trend is observed (Fig. 5). Several genes including *Gas2, Dclk1* and *Rpl38* have high expression in several SV cell types. The expression pattern of non-reduced EP genes in the stria does not seem to be impacted by sex or age.

### Reduced EP Genes are Also Expressed in Other Cells of the Cochlear Duct

**Fig. 6:**
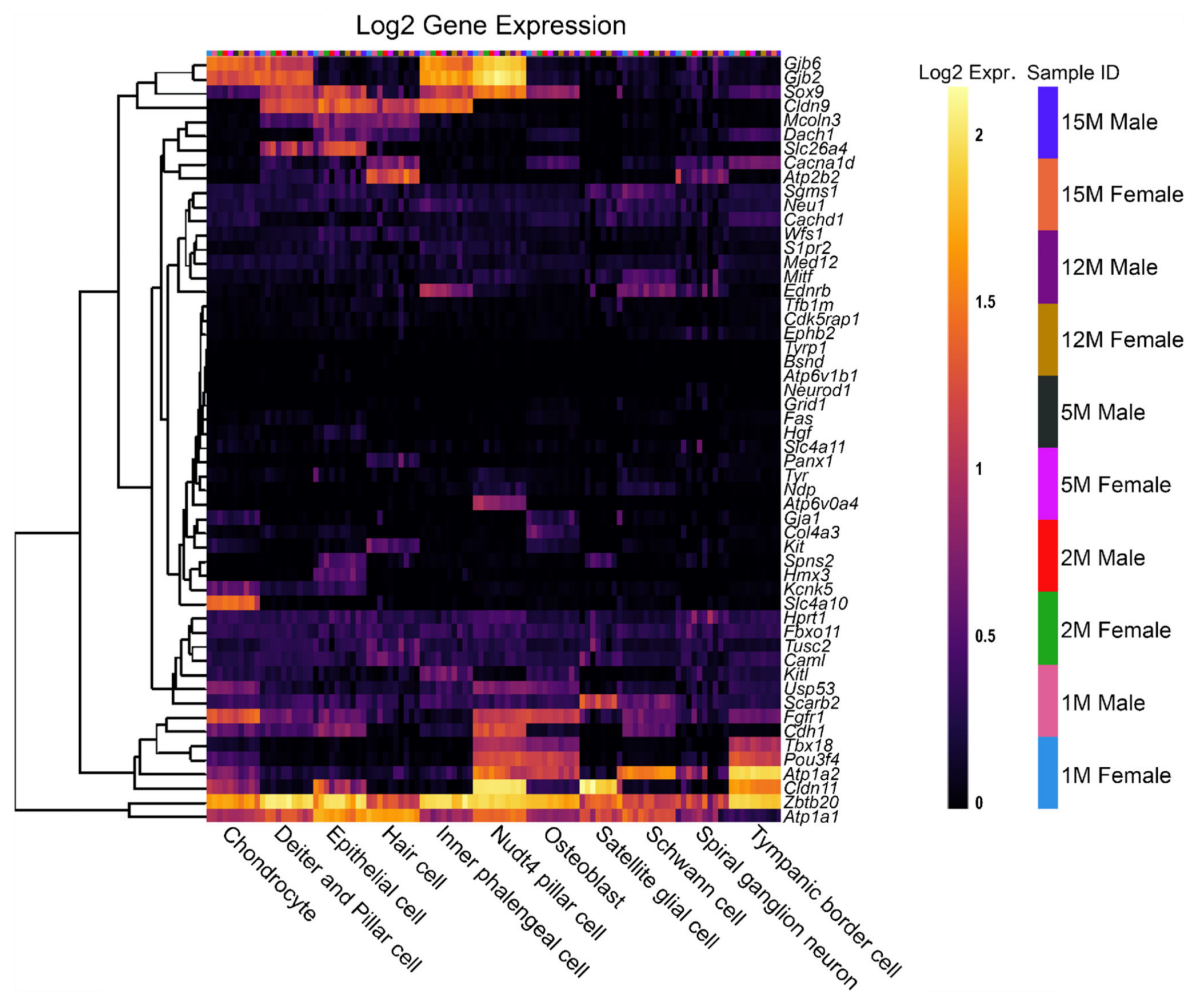
The expression of reduced EP genes in organ of Corti cell types between 1 and 15 months of age. The 55 reduced EP genes were the input for the multi-gene array. The housekeeping gene *Hprt1* was added to the gene list for comparison. Settings were adjusted so that the primary grouping was cell type. Genes were clustered based on a Manhattan (city block) approach. Within each gene and cell-type category, samples are presented in increasing age from left to right, with females and males within the same age group adjacent to each other. Source: UMgEAR Portal, [14]

**Fig. 7:**
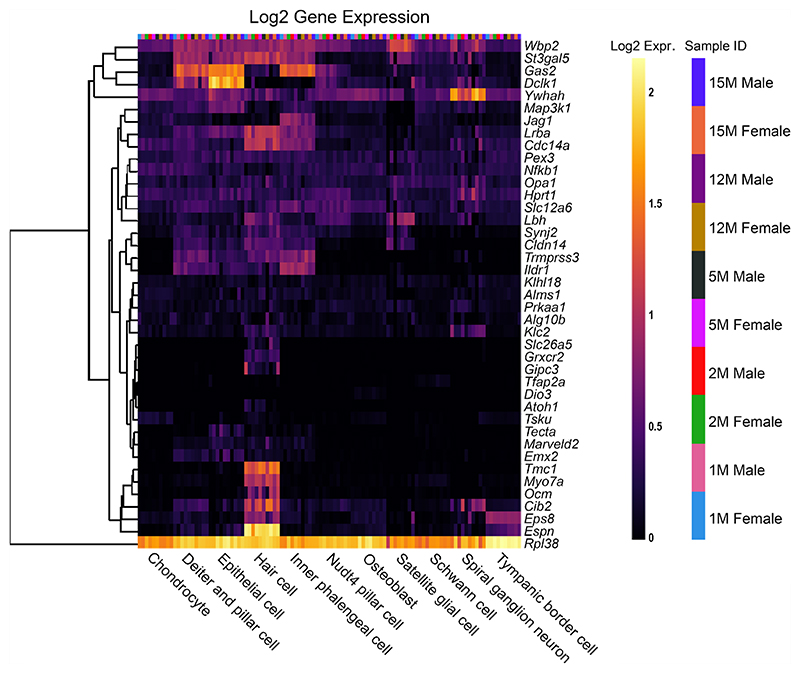
The expression of non-reduced EP genes in organ of Corti cell types between 1 and 15 months of age. The 43 non-reduced EP genes were the input for the multi-gene array. The housekeeping gene *Hprt1* was included for comparison. The primary grouping was set to cell type and genes were clustered based on a Manhattan (city block) approach. Within each gene and cell-type category, samples are presented in increasing age from left to right, with females and males within the same age group adjacent to each other. Source: UMgEAR Portal, [14]

To investigate the pattern of expression in cell types of the organ of Corti, both reduced and non-reduced EP genes were analysed using an organ of Corti dataset [14]. Interestingly, there are a large proportion of reduced EP genes with high expression in the organ of Corti (Fig. 6), particularly in *Nudt4*-positive pillar cells and osteoblasts. For some genes such as *Atp2b2* and *Atp6v0a4*, expression in the organ of Corti is high despite very low expression in the SV cell types. This highlights that disruption to gene expression in any cell type of the cochlea involved in potassium recycling could lead to reduced EP [27]. The expression of reduced EP genes in the organ of Corti does not seem to be sex- or age-dependent. The non-reduced EP gene list also was analysed using this dataset (Fig. 7). Many non-reduced EP genes are expressed in the organ of Corti, particularly in hair cells. However, there appears to be less expression of non-reduced EP genes in the organ of Corti compared to reduced EP genes. This analysis highlights that cell types outside of the lateral wall are important to producing and maintaining EP.

### Reduced EP Genes can be Categorised into a Broad Range of Molecular Functions and Biological Processes

**Fig. 8:**
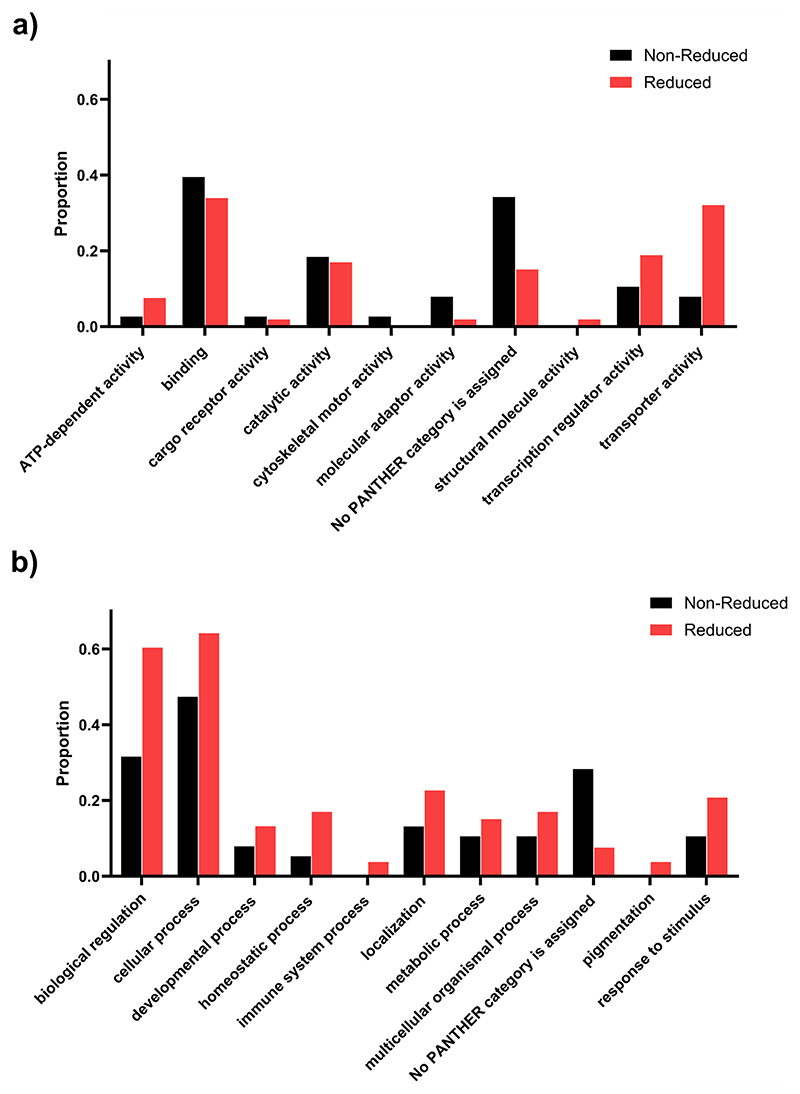
The molecular function and biological process categories of EP genes following GOSlim analysis. **a** The molecular function categories for non-reduced (black) and reduced (red) EP genes. **b** The biological function categories for non-reduced (black) and reduced (red) EP genes. The proportion of genes in each group with the high-level GO terms listed are plotted for each of the sub-categories

To investigate the function of the reduced EP genes and search for common pathways, a GOSlim analysis (high-level version of Gene Ontology) was used to investigate enriched categories of molecular processes (Fig. 8a) or biological function (Fig. 8b) of the genes in the reduced EP (n = 55) list compared to those in the non-reduced EP gene list (n = 43). There is a broad range of categories of molecular function for reduced EP genes. The highest proportion of reduced EP genes is in the binding sub-category (0.34) which is similar to that of non-reduced EP genes (0.39). However, it is notable that the reduced EP genes have a much larger proportion of genes in both the transporter activity and transcription regulator activity (0.32 and 0.19) compared to non-reduced EP genes (0.08 and 0.11 respectively). This aligns with the known key functions required for generating EP [3].

There is also a varied range of broad categories of biological processes of reduced EP genes (Fig. 8b). The proportion of reduced EP genes in each biological process sub-category is higher than that of non-reduced EP genes in almost all categories, particularly for biological regulation where the proportion almost doubles (0.6 compared to 0.32) and for cellular process (0.64 compared to 0.47).

### There is a Large Degree of Variation in Wildtype EP Values

**Fig. 9:**
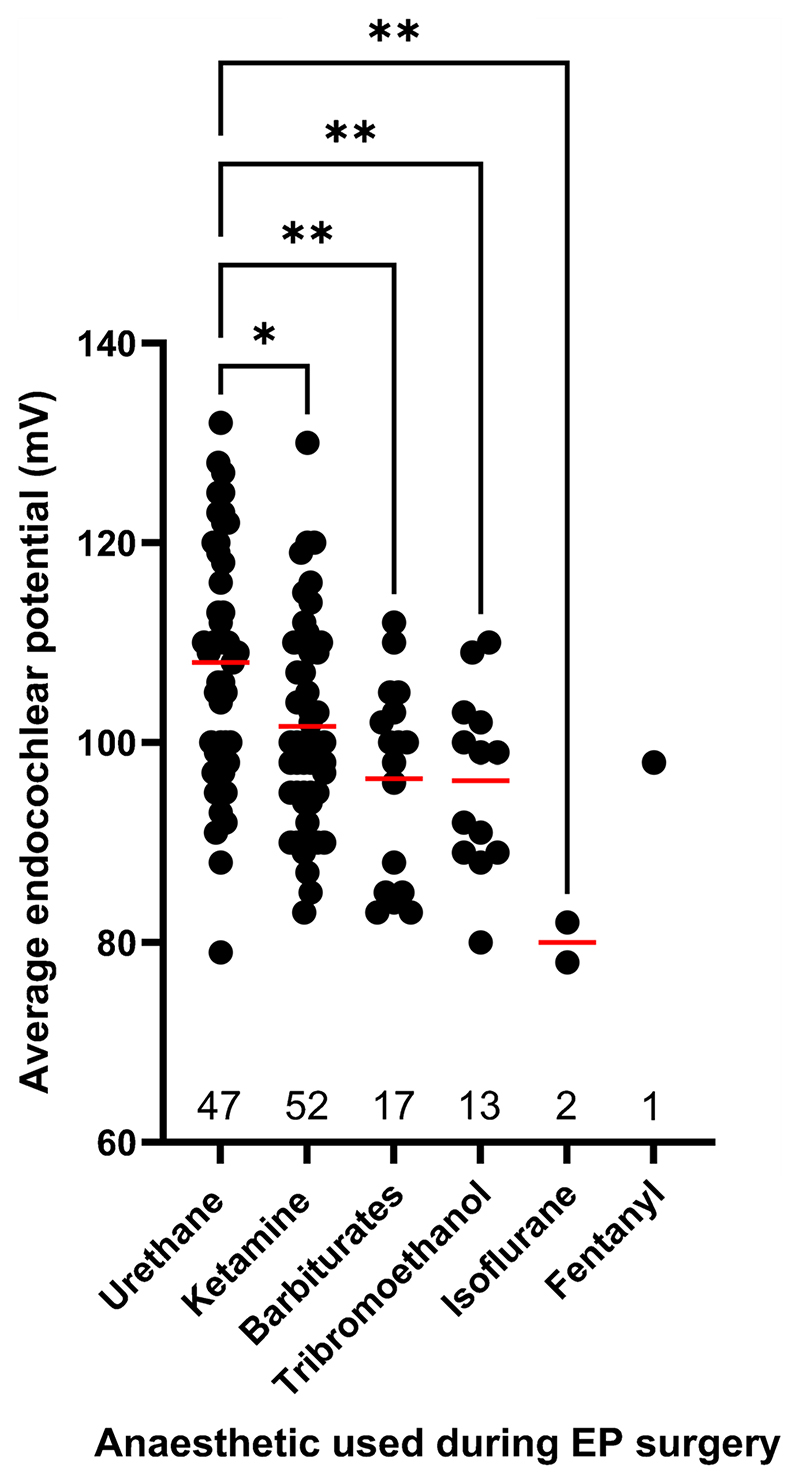
The effect of the anaesthetic agent on the EP value of wildtype control mice. Each point represents the mean control EP level in young adults from a single publication from the original searches, as given in Supplementary Table 1, and datasets represent a variety of genetic backgrounds and both sexes. Datasets were tested for normality using a Shapiro–Wilk test and for equality of variance using a Brown-Forsythe test. Following this, a one-way ANOVA test was performed on the dataset. The mean rank of each column was compared with the mean rank of every other column for multiple comparisons * *p* < 0.05, ** *p* < 0.01. Data are presented as individual datapoints for each dataset with the mean bar overlaid (red). The total number of values for each anaesthetic agent are presented along the x-axis

It was noted during creation of the gene list that there was a large degree of variation in the control EP values reported across papers. To test whether the anaesthetic agent used was a significant contributing factor to this variation, we plotted EP of the control group (mV) against the anaesthetic agent used during surgery (Fig. 9; Supplementary Table S1). Urethane anaesthetic generated a significantly higher average wildtype EP value compared to all other anaesthetic drug classes. Ketamine generated the second highest average wildtype EP value.

## Discussion

In this review we collate a list of 55 genes known to be involved in reduced EP in mice. We also generated a list of 43 genes for which deafness and normal EP values are reported (non-reduced EP genes). We show that 27 of the reduced EP genes have been associated with deafness in the human population and suggest that these genes are likely to be associated with reduced EP in human patients.

Using a systematic approach such as PRISMA was helpful for defining specific query terms to apply to large databases such as Medline and Embase for retrieving literature. However, we observe that these databases did not have access to full text searching during the multipurpose search function and therefore missed several papers where EP was recorded but key findings not described in the abstract. This was particularly disadvantageous when searching for negative findings reported in the non-reduced EP gene list where the results were often not described in the abstract. There is no clear way to circumvent this problem when searching for literature in such databases, and even when full-text searching is available some publications will still be inaccessible due to paywalls. To address this limitation, we used three additional methods for retrieving literature to include in this review to ensure genes were not missed from the final gene lists. The most comprehensive lists of genes with reports of EP measurements came from interrogating a manually curated list of genes involved in deafness.

Using AI LLMs as an efficiency tool during the systematic review process is becoming an increasingly tempting process. This review made use of AI LLMs as a tool to retrieve literature for inclusion in the review. All sources were subsequently manually verified before inclusion into the EP gene lists. We report a high degree (63%) of hallucinations generated during literature retrieval, in addition to the genes that were not discovered using this method. An important message from undertaking this review is that publicly available AI is currently not equipped to perform literature reviews accurately, and manual checking of all information provided is critical due to the high false positive rate. These conclusions are supported by another review which highlights the lack of referencing and production of verifiable sources when asked for factual information [28]. Another paper reports up to 82% accuracy during the article screening process [29] which contrasts greatly with our 63% inaccuracy rate. However, designing prompts for including vs excluding articles is a very different application of LLMs to searching for literature to include for review in the first instance. We also highlight that using a multi-pronged approach to systematic searching provides the highest chance of capturing the full scope of papers of interest. No single approach retrieved the full list of EP genes, so it is beneficial to use multiple search approaches and databases to retrieve relevant papers before analysing the results of a systematic review.

Analysis of the reduced EP gene list revealed a plethora of cell type-specific expression within the stria vascularis and in the cochlea. Importantly, we highlight that cell types in the SV are not the only sites within the ear that are important for maintaining EP. Gene expression for several reduced EP genes was high in the organ of Corti. This point has been highlighted in previous literature [27], and this review adds to this by demonstrating the broad range of cell types in which reduced EP genes are highly expressed, particularly outside of the lateral wall. We also report that reduced EP genes are involved in a wide range of molecular pathways and biological functions, and this is not limited to the function of ion channels. However, we note that the annotated categories for genes in PANTHER (Protein ANalysis THrough Evolutionary Relationships) are curated from comparisons of sequence data and therefore categories are general and lack specificity to the inner ear.

During annotation of the reduced EP gene list it was noted that the wildtype EP value varied significantly, making it difficult to compare the extent of EP reduction across studies. We analysed the effect of anaesthetic agent on wildtype EP value reported in papers and found that urethane produced significantly higher EP values compared to all other anaesthetic agents, likely due the depressive effect that some other anaesthetics can exert on the cardiovascular system [30]. Interestingly, within each anaesthetic agent, there was a lot of variation of wildtype EP values. This could be partially explained by the range of doses which are reported across papers. However, variation may also arise from other factors, for example, the age of the control mice or genetic background of the strain being used, as both have been reported to have an impact on EP level [31]. One paper reports a clear example of this where the wildtype EP in C57BL/6 mice was on average approximately 15 mV higher than the wildtype EP in 129 Sv mice of the same age [32]. The *Cx26*^*S17F*^ mutation that was investigated produced a larger reduction in EP on the 129 Sv genetic background compared to C57BL/6 further highlighting the importance of interpreting results in the context of the study [32]. Genomic variants occurring naturally in inbred strains have been reported to be associated with different EP levels, such as between C57BL/6 J and BALB/cJ mice [33, 34]. Variation could also arise from differences in the EP recording setup such as variable amplifier settings, microelectrode properties (impedance) or route of access to scala media via the lateral wall or through the organ of Corti. EP can also vary depending on the turn of the cochlear duct [35].

Finally, there is increasing interest in developing new treatments for specific types of hearing loss following the recent successes of gene therapy in children with *OTOF* mutations [36–38]. Treatments aimed at hair cells or their synapses (like *OTOF* gene therapy) are more likely to be successful if it is known that their EP should be normal, as in the human diseases listed in Table 2, where mutant mice have normal EPs. On the other hand, people affected by mutations in the reduced EP gene list may be candidates for generic treatments aimed at boosting strial function by stimulating ion pumping activity or blood flow for example. The lists presented here can inform the direction of translational research for different forms of hearing loss.

## Supplementary Material

Supplementary Information The online version contains supplementary material available at https://doi.org/10.1007/s10162-026-01046-y.

Supplementary Table

## Figures and Tables

**Table 1:** The full list of 55 genes with experimental evidence showing reduced EP in mice when gene expression was disrupted. Key information highlighted includes gene name and Ensembl ID, known link to human deafness cases as well as a reference to a paper in which EP was recorded

Gene Name	Ensembl ID	Link to human deafness	Refer-ence for EP
*Atpla1*	ENSMUSG00000033161	Autosomal dominant axonal Charcot-Marie-Tooth disease type 2DD	[39]
*Atp1a2*	ENSMUSG00000007097	No	[39]
*Atp2b2*	ENSMUSG00000030302	Autosomal dominant 82 deafness, autosomal recessive 12 deafness,	[40]
*Atp6v0a4*	ENSMUSG00000038600	Distal renal tubular acidosis 3, with or without sensorineural hearing loss	[41]
*Atp6v1b1*	ENSMUSG00000006269	Distal renal tubular acidosis 2 with progressive sensorineural hearing loss	[42]
*Bsnd*	ENSMUSG00000025418	Bartter syndrome, type 4a; Sensorineural deafness with mild renal dysfunction	[43]
*Cachd1*	ENSMUSG00000028532	No	[44]
*Cacna1d*	ENSMUSG00000015968	Primary aldosteronism, seizures, and neurologic abnormalities; Sinoatrial node dysfunction and deafness	[45]
*Cam1*	ENSMUSG00000021501	No	[46]
*Cdh1*	ENSMUSG00000000303	No	[47]
*Cdk5rap1*	ENSMUSG00000027487	No	[48]
*Cga*	ENSMUSG00000028298	No	[49]
*Cldn11*	ENSMUSG00000037625	No	[50]
*Cldn9*	ENSMUSG00000066720	Autosomal recessive 116 deafness	[51]
*Col4a3*	ENSMUSG00000079465	Alport syndrome 3 A, autosomal dominant; Alport syndrome 3B, autosomal recessive; Hematuria, benign familial, 2	[52]
*Dach1*	ENSMUSG00000055639	No	[53]
*Ednrb*	ENSMUSG00000022122	No	[54]
*Ephb2*	ENSMUSG00000028664	No	[55]
*Fas*	ENSMUSG00000024778	No	[56]
*Fbxo11*	ENSMUSG00000005371	No	[57]
*Fgfr1*	ENSMUSG00000031565	Pfeiffer syndrome; Jackson-Weiss syndrome; Osteoglophonic dysplasia; Trigonocephaly 1; Hypogonadotropic hypogonadism 2 with or without anosmia; Hartsfield syndrome	[58]
*Gja1*	ENSMUSG00000050953	Craniometaphyseal dysplasia, autosomal recessive; Erythrokeratodermia variabilis et progressiva 3; Oculodentodigital dysplasia AD; Oculodentodigital dysplasia, autosomal recessive; Palmoplantar keratoderma with congenital alopecia; Syndactyly, type III; some reported with conductive hearing loss	[59]
*Gjb2*	ENSMUSG00000046352	Deafness, autosomal dominant 3 A; Deafness, autosomal recessive 1 A; Hystrix-like ich-thyosis with deafness; Keratitis-ichthyosis-deafness syndrome; Keratoderma, palmoplantar, with deafness; Vohwinkel syndrome; Bart-Pumphrey syndrome	[60]
*Gjb6*	ENSMUSG00000040055	Deafness, autosomal dominant 3B; Deafness, autosomal recessive 1B; Deafness, digenic GJB2/GJB6; Ectodermal dysplasia 2, Clouston type	[61]
*Grid1*	ENSMUSG00000041078	No	[62]
*Hgf*	ENSMUSG00000028864	Deafness, autosomal recessive 39	[63]
*Hmx3*	ENSMUSG00000040148	No	[16]
*Kcnk5*	ENSMUSG00000023243	No	[64]
*Kit*	ENSMUSG00000005672	No	[65]
*Kit1*	ENSMUSG00000019966	Waardenburg syndrome, type 2 F; Deafness, autosomal dominant 69, unilateral or asymmet-ric; Hyperpigmentation with or without hypopigmentation; Skin/hair/eye pigmentation 7, blond/brown hair	[66]
*Mcoln3*	ENSMUSG00000036853	No	[67]
*Med12*	ENSMUSG00000079487	Hardikar syndrome; Lujan-Fryns syndrome; Ohdo syndrome, X-linked; Opitz-Kaveggia syndrome	[68]
*Mitf*	ENSMUSG00000035158	Waardenburg syndrome, type 2 A; Tietz albinism-deafness syndrome; COMMAD syn drome; Melanoma, cutaneous malignant, susceptibility to, 8	[69]
*Ndp*	ENSMUSG00000040138	Norrie disease; Exudative vitreoretinopathy 2, X-linked	[70]
*Neu1*	ENSMUSG00000007038	Sialidosis, type I; Sialidosis, type II	[71]
*NeuroD1*	ENSMUSG00000034701	No	[72]
*Panx1*	ENSMUSG00000031934	No	[73]
*Pou1f1*	ENSMUSG00000004842	No	[74]
*Pou3f4*	ENSMUSG00000056854	Deafness, X-linked 2	[75]
*S1pr2*	ENSMUSG00000043895	Deafness, autosomal recessive 68	[76]
*Scarb2*	ENSMUSG00000029426	No	[77]
*Sgms1*	ENSMUSG00000040451	No	[78]
*Slc26a4*	ENSMUSG00000020651	Pendred syndrome; Deafness, autosomal recessive 4, with enlarged vestibular aqueduct	[79]
*Slc4a10*	ENSMUSG00000026904	No	[80]
*Slc4a11*	ENSMUSG00000074796	Corneal endothelial dystrophy and perceptive deafness; Corneal endothelial dystrophy, auto-somal recessive; Corneal dystrophy, Fuchs endothelial, 4	[81]
*Sox9*	ENSMUSG00000000567	Campomelic dysplasia with autosomal sex reversal; Campomelic dysplasia (some with hear-ing loss); Acampomelic campomelic dysplasia; 46XY sex reversal 10; 46XX sex reversal 2	[82]
*Spns2*	ENSMUSG00000040447	Deafness, autosomal recessive 115	[83]
*Tbx18*	ENSMUSG00000032419	No	[84]
*Tfb1m*	ENSMUSG00000036983	No	[85]
*Tusc2*	ENSMUSG00000010054	No	[86]
*Tyr*	ENSMUSG00000004651	No	[87]
*Tyrp1*	ENSMUSG00000005994	No	[88]
*Usp53*	ENSMUSG00000039701	Cholestasis, progressive familial intrahepatic, 7, with or without hearing loss	[89]
*Wfs1*	ENSMUSG00000039474	Deafness, autosomal dominant 6/14/38; Wolfram syndrome 1; Wolfram-like syndrome, autosomal dominant;	[90]
*Zbtb20*	ENSMUSG00000022708	Primrose syndrome	[91]

**Table 2:** The full list of 43 genes with experimental evidence showing non-reduced EP in mice when gene expression was disrupted. Key information highlighted includes gene name and Ensembl ID, known link to human deafness cases as well as a reference to a paper in which EP was recorded

Gene Name	Ensembl ID	Link to human deafness	Reference for EP
*Alg10b*	ENSMUSG00000075470	Susceptibility to long QT syndrome 2, with *KCNH2*	[92]
*Alms1*	ENSMUSG00000063810	Alstrom syndrome	[93]
*Atoh1*	ENSMUSG00000073043	Deafness, autosomal dominant 89	[94]
*Cdc14a*	ENSMUSG00000033502	Deafness, autosomal recessive 32, with or without immotile sperm	[95]
*Cib2*	ENSMUSG00000037493	Deafness, autosomal recessive 48	[96]
*Cldn14*	ENSMUSG00000047109	Deafness, autosomal recessive 29	[97]
*Dclk1*	ENSMUSG00000027797	No	[98]
*Dio3*	ENSMUSG00000075707	No	[99]
*Emx2*	ENSMUSG00000043969	Schizencephaly	[100]
*Eps8*	ENSMUSG00000015766	Deafness, autosomal recessive 102	[101]
*Espn*	ENSMUSG00000028943	Deafness, autosomal recessive 36; Deafness, neurosensory, without vestibular involvement, autosomal dominant; Usher syndrome, type 1 M	[102]
*Gas2*	ENSMUSG00000030498	Deafness, autosomal recessive 125	[103]
*Gipc3*	ENSMUSG00000034872	Deafness, autosomal recessive 15	[104]
*Grxcr2*	ENSMUSG00000073574	Deafness, autosomal recessive 101	[105]
*Ildr1*	ENSMUSG00000022900	Deafness, autosomal recessive 42	[106]
*Jag1*	ENSMUSG00000027276	Alagille syndrome 1; Charcot-Marie-Tooth disease, axonal, type 2HH; Deafness, congenital heart defects, and posterior embryotoxon	[107]
*Klc2*	ENSMUSG00000024862	Spastic paraplegia, optic atrophy, and neuropathy, SPOAN syndrome	[108]
*Klhl18*	ENSMUSG00000054792	No	[109]
*Lbh*	ENSMUSG00000024063	No	[110]
*Lrba*	ENSMUSG00000028080	Immunodeficiency, common variable, 8, with autoimmunity	[111]
*Map3k1*	ENSMUSG00000021754	46XY sex reversal 6; DFNB128?	[112]
*Marveld2*	ENSMUSG00000021636	Deafness, autosomal recessive 49	[113]
*Minar2*	ENSMUSG00000050875	Deafness, autosomal recessive 120	[114]
*Mir96*	ENSMUSG00000065586	Deafness, autosomal dominant 50	[115]
*Myo7a*	ENSMUSG00000030761	Usher syndrome, type 1B; Deafness, autosomal recessive 2; Deafness, autosomal dominant 11	[116]
*Nfkb1*	ENSMUSG00000028163	Immunodeficiency, common variable, 12	[117]
*Ocm*	ENSMUSG00000029618	No	[118]
*Opa1*	ENSMUSG00000038084	Optic atrophy 2, X-linked	[119]
*Pex3*	ENSMUSG00000019809	Peroxisome biogenesis disorder 10 A, Zellweger	[120]
*Prkaa1*	ENSMUSG00000050697	No	[121]
*Prop1*	ENSMUSG00000044542	Pituitary hormone deficiency, combined, 2	[122]
*Rpl38*	ENSMUSG00000057322	No	[123]
*Slc12a6*	ENSMUSG00000027130	Charcot-Marie-Tooth disease, axonal, type 2II; Agenesis of the corpus callosum with peripheral neuropathy	[124]
*Slc26a5*	ENSMUSG00000029015	Deafness, autosomal recessive 61	[118]
*St3gal5*	ENSMUSG00000056091	Salt and pepper developmental regression syndrome	[125]
*Synj2*	ENSMUSG00000023805	No	[126]
*Tecta*	ENSMUSG00000037705	Deafness, autosomal recessive 21; Deafness, autosomal dominant 8/12	[127]
*Tfap2a*	ENSMUSG00000021359	Branchiooculofacial syndrome	[128]
*Tmc1*	ENSMUSG00000024749	Deafness, autosomal recessive 7; Deafness, autosomal dominant 36	[129]
*Tmprss3*	ENSMUSG00000024034	Deafness, autosomal recessive 8/10	[130]
*Tsku*	ENSMUSG00000049580	No	[131]
*Wbp2*	ENSMUSG00000034341	Deafness, autosomal recessive 107	[132]
*Ywhah*	ENSMUSG00000018965	No	[133]

## Data Availability

All publications used in this review are available online. Gene expression data were obtained from the gEAR expression database available online. EP values used for Fig. 9 are presented in Supplementary Table 1, along with the references.
